# Establishment of sheep nasal mucosa explant model and its application in antiviral research

**DOI:** 10.3389/fmicb.2023.1124936

**Published:** 2023-05-15

**Authors:** Jian Zheng, Jian Lin, Yichao Ma, Chengjie Yang, Qiu Zhong, Yuchen Li, Qian Yang

**Affiliations:** MOE Joint International Research Laboratory of Animal Health and Food Safety, College of Veterinary Medicine, Nanjing Agricultural University, Nanjing, Jiangsu, China

**Keywords:** nasal mucosa explant, sheep, *Bacillus subtilis*, pseudorabies virus, ISG15

## Abstract

The nasal mucosa is the first barrier to pathogen invasion through the respiratory tract. Few studies have focused on nasal resistance to invasion by respiratory pathogens due to the lack of models related to the nasal mucosa. Hence, it is necessary to construct a nasal mucosal model to study host-pathogen interactions. We established a long-term *in vitro* sheep nasal mucosa explant model (NMEM), which exhibited typical epithelial cilia and epithelial proliferation ability within 11 days. Moreover, to evaluate whether the NMEM was suited for *in vitro* pathogenic study, we used pseudorabies virus (PRV) and showed that it successfully infected and produced severe lesions in the NMEM, particularly interferon (*IFN*)-stimulated gene product 15 (*ISG15*). IFN decreased significantly after the PRV infection. Similarly, we used this NMEM model to screen several antiviral substances, such as probiotics and drugs. A previous study showed that nasal commensal bacteria, particularly *Bacillus subtilis*, had high antiviral activity. Then, we used the NMEM to evaluate six sheep-derived *B. subtilis* strains and demonstrated that it significantly induced the production of IFN and expression of ISG15. The sheep-derived *B. subtilis* was pretreated with the sheep NMEM before the PRV infection to evaluate the antiviral effect. The results showed that NSV2 significantly inhibited infection by PRV and reduced the viral load (*p* < 0.05). Furthermore, NSV2 may inhibit PRV replication by enhancing ISGylation of cells. In conclusion, we established a reliable *in vitro* culture model of sheep NMEM, and applied it in antiviral research.

## 1. Introduction

As the gateway of the respiratory tract, the nasal cavity is in direct contact with the external environment, and is vulnerable to invasion and colonization by pathogenic microorganisms (Erturk-Hasdemir and Kasper, 2013). However, it is difficult to investigate the interaction between pathogenic microorganisms in the nasal cavity and the infection mechanism. Cells and animals are the two most commonly used research models to explore the interaction between a pathogen and its host. However, while conventional *in vitro* studies of single-cell cultures are highly instrumental for elucidating the virus-host interaction at the cellular level, they do not reflect the broad natural tropism (including myeloid lineage, epithelial, endothelial, and fibroblast cells) or mechanism underlying the spread of pathogenic microorganisms *in vivo* (Sinzger et al., 2008; Gerna et al., 2017). Animal models are expensive; for example, the price of a cynomolgus monkey has exceeded USD 20,000 since COVID-19. Animal experiments require adequately equipped laboratories and qualified personnel (Russo et al., 2016). More importantly, animals are living creatures that deserve respect and should not be wasted for any purpose. This is why the 3Rs principles of Replacement, Reduction, and Refinement are crucial (Diaz et al., 2020). To adhere to these principles, we require reliable *in vitro* nasal mucosa experimental models.

The nasal mucosa is a complex tissue that interacts with the environment and influences local and systemic changes. The nasal mucosa is composed of the epithelial cell layer, basement membrane layer, submucosa, and a large number of blood vessels and glands. Models of the nasal cavity include cell-submerged culture models (Devalia and Davies, 1991), air-liquid culture models (Muller et al., 2013; Aydin et al., 2021), nasal mucosa organoids models (Liu et al., 2021; Chiu et al., 2022), and nasal mucosa explant culture models (Alfi et al., 2020). An explant model not only simulates the entire mucosal structure *in vivo*, but also preserves the interaction between different cells, making it suitable for *in vitro* etiological research (Tulinski et al., 2013). Nasal mucosa explants of humans (Alfi et al., 2020), pigs (Glorieux et al., 2011), rats (Fanucchi et al., 1999), cows (Steukers et al., 2012), and cat (Kruger et al., 2021; Farber et al., 2022) have been cultured and studied. Furthermore, the characteristics of infections with different pathogenic microorganisms have been investigated in respiratory explants (Van Poucke et al., 2010; Lamote et al., 2016; Di Teodoro et al., 2018). However, these explant models were not systematically evaluated or tested, so may not have been uniform in terms of mucosal tissue size. Moreover, their culture times were short, which is associated with low reliability.

A large number of commensal microbiotas are detectable in the nasal cavity, which plays an important role in maintaining the health of the respiratory tract (Thaiss et al., 2016; Man et al., 2017; Kim et al., 2019). The nasal mucosa explant model (NMEM) has important potential to analyze the functions of nasal commensal microbiota. Previous studies have shown that *Bacillus subtilis* significantly enhances the innate immunity of the nasal mucosa (Yang et al., 2018), as well as resistance to pathogenic microorganism infection (Yang et al., 2018; Yuan et al., 2018; Guo et al., 2020; Kuebutornye et al., 2020; Taggar et al., 2021). As an aerobic or facultative anaerobic bacterium, *Bacillus subtilis* induces highly effective nasal mucosal immunity (Kovacs, 2019). However, those studies only used exogenous *Bacillus subtilis*, rather than nasal commensal *Bacillus subtilis*, so were unable to examine internal effects.

The innate immune system in the nasal cavity can inhibit the infection of respiratory pathogenic microorganisms by producing interferon (IFN), which is a first-line defense mechanism to maintain respiratory health (Garcia-Sastre and Biron, 2006; Cheon et al., 2013; Kim et al., 2013). Interferon-stimulated genes (ISGs) are essential for the antiviral function of IFNs. Various studies have demonstrated that ISG15, 2′-5′ oligoadenylate synthase (OAS2), double-stranded RNA protein kinase R (PKR), interferon-induced transmembrane protein 1 (IFITM1), and nitric oxide synthase (NOS) play a crucial role in inhibiting viral infections (Knapp et al., 2003; Lowenstein and Padalko, 2004; Silverman, 2007). ISGs can act on multiple stages of virus infection, such as invasion, shelling, replication, assembly, and release (Gonzalez-Sanz et al., 2016; Liu et al., 2018). As a result, microorganisms have the potential to play a critical role in preventing virus invasion and infection by regulating the nasal innate immune system.

This study constructed a long-term cultured sheep NMEM, which had good *in vitro* activity and simulated the infection characteristics *in vivo*. The role and potential function of nasal commensal *Bacillus subtilis* in the sheep nasal cavity was explored using this model.

## 2. Methods

### 2.1. Cells, virus and reagents

VERO (ATCC®CRL-1586) and PK-15 cells (ATCC®CCL-33) were cultured in Dulbecco’s modified Eagle’s medium (CORNING, USA) supplemented with 10% fetal bovine serum (Gibco) at 37°C in a humidified atmosphere containing 5% CO2. The PRV strain ZJ 01 was isolated from a pig herd at China in 2012 (Gu et al., 2015), which was used for all experiments. The PRV were proliferated in PK-15 cells and stored at −80°C. *β-Streptococcus equinus strain SheepZ001* were cultured in Tryptone soybean broth (TSB) + 0.05% fetal bovine serum (FBS) medium at 37°C, 220 rpm. *Pasteurella multocida HB01* (Serotype A) were cultured in Brain-heart extract broth (BHI) medium at 37°C, 220 rpm. *Bacillus subtilis 168*, *WB800* and other *Bacillus subtilis* group isolated from sheep nasal cavity were cultured in Luria-Bertani (LB) medium at 37°C, 220 rpm. Anti-PRV gB-protein monoclonal antibodies (1B1, prepared and stored in our laboratory), anti-GAPDH antibody (Proteintech), anti-cytokeratin18 (Abcam), anti-claudin 1(Abcam), anti-caspase3 (Proteintech); anti-caspase9 (Proteintech); anti-ISG15 antibody (Abcam), anti-PCNA antibody (Abcam), anti-CD3 antibody (Dako), anti-IgA antibody (Abcam) and anti-CD208 antibody (Dendritics) was used in the study.

### 2.2. Isolation and cultivation of the sheep nasal mucosa explants

**Figure 1:**
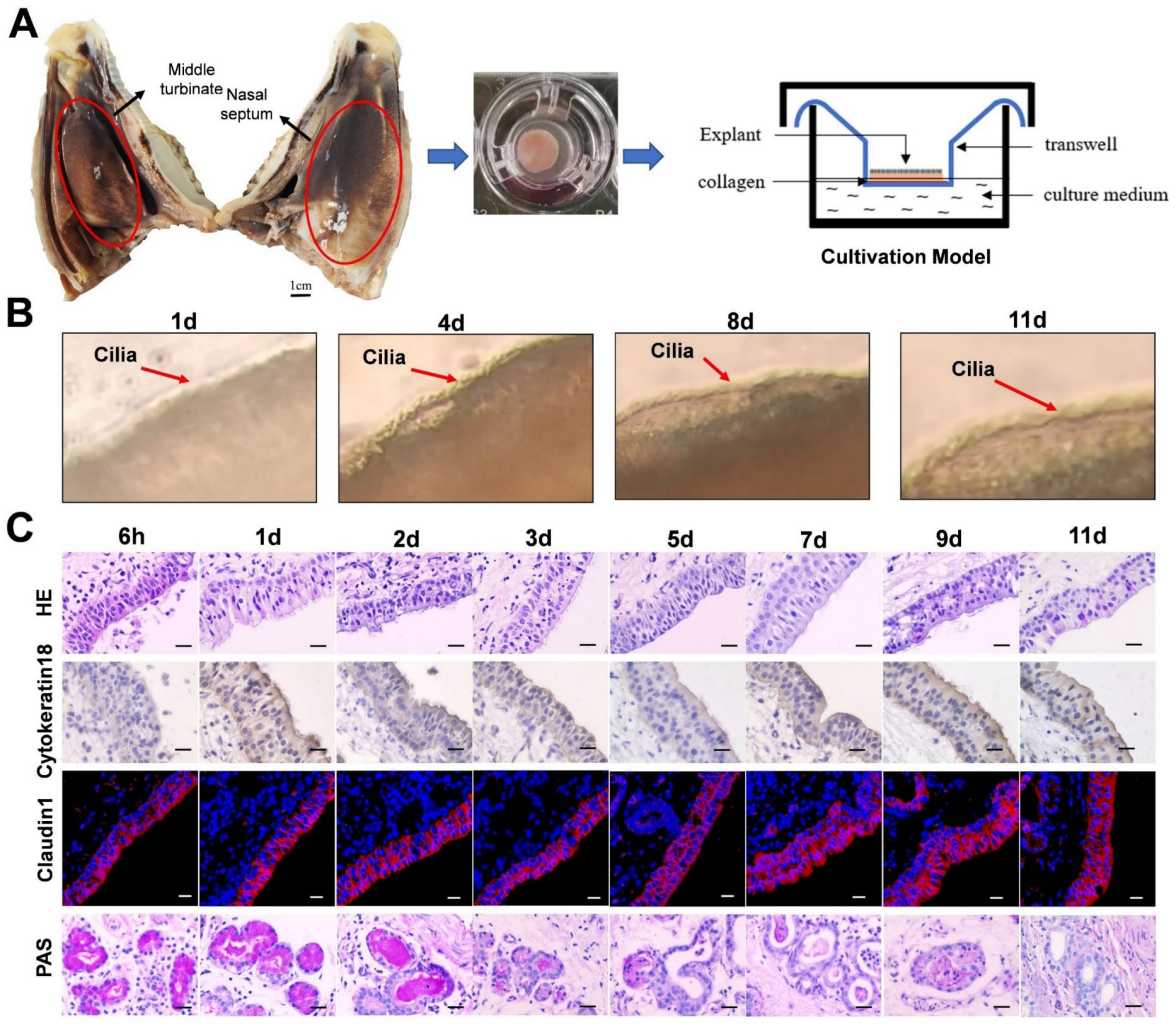
The establishment of sheep nasal mucosa explant culture models. **(A)** Construction of the air-liquid culture model of sheep nasal mucosa explants. **(B)** The cilia wiggling of sheep nasal mucosa explants were observed under light microscope. **(C)** HE staining for evaluate of the structure characteristics. The expression characteristics of cytokeratin18 were observed by immunohistochemistry. The Claudin1 was used to evaluate the integrity of epithelial barrier by immunofluorescence. The PAS was used to observed the secretory characteristics of the subcutaneous glands. The nasal mucosa explants of sheep at different times were obtained from three mucosal tissues of three sheep. Scar bar = 50 μm.

The healthy 6-month-old lambs (*n* = 3) were euthanized by intravenous injection of pentobarbital sodium (100 mg/kg). After death, lambs were decapitated, the different part of nasal cavity (nasal concha and septum) was immersed into a PBS medium (5% double antibody, 1% amphotericin and gentamicin) (Figure 1A). Next, the different of nasal cavity were transferred into a 50 ml centrifuge tube filled with PBS and antibiotics. After shaking the tube, we washed it 3–4 times, with each wash lasting 5–10 min. Once complete, we transferred the washed nasal tissue onto a sterile plate. Using ophthalmic scissors and tweezers, we gently peeled off the nasal mucosa tissue. In addition, we extract a piece of nasal mucosa and place it on a new sterile plate with the surface facing upwards. Then, nasal mucosa is processed into uniform circular tissue blocks measuring 8 mm in diameter using a tissue sampler. The nasal cavity is a complex structure with varying thicknesses of mucosa and epithelial characteristics throughout its different parts. To ensure consistency, we chose to focus on the mucosa of the inferior nasal concha in the respiratory area of the nasal cavity. On average, about 40 pieces of mucosal tissue can be obtained from nasal cavity of 6-month-old sheep. The mucosal tissue was pretreated and placed onto a 12-hole transwell device with the mucosal surface facing upwards. The upper chamber filter membrane had a pore diameter of 0.4 μM. Approximately 600 μL of culture medium (Alfi et al., 2020) was added to the entire transwell device, which was only half the thickness of the mucosal tissue. The tissue was then cultured at 37°C and 5% CO2, with the medium being replaced daily.

### 2.3. Observation of cilia oscillations

The nasal mucosal explants were fold at different cultured times, make the cilia face outward. Then, the folded position was observed by optical microscope, and record by video.

### 2.4. Immunohistochemistry

The nasal mucosal explants were fixed in a phosphate-buffered 4% formaldehyde solution for 24 h. After fixation, the samples were embedded in paraffin. Sections (5 mm thick) were cut, deparaffinized in xylene, rehydrated in descending grades of alcohol. For immunohistochemistry analysis, antigen retrieval was performed for 30 min with citrate buffer at pH 6.0 in a Decloaking Chamber at 95°C. Slides were blocked with 5% bovine serum albumin (BSA) 1 h and then incubated with anti-cytokeratin18 (1: 200) overnight at 4°C in a humidified chamber. The SABC-POD Kit was used for the amplification and visualization of the signal.

### 2.5. Immunofluorescence

Sections were rinsed and subjected to antigen demasking as described above. After were treated with 5% BSA for 1 h and incubated with mouse anti-Claudin1 (1: 200), anti-PCNA, anti-CD3 (1:200), anti-IgA (1:200) and anti-CD208 (1:100) overnight at 4°C, followed by incubation with fluorescent secondary antibodies for 1 h at 25°C. Cell nuclei were stained by incubation with diamidino-2-phenylindole (DAPI) for 5 min, and observed under a confocal laser microscope (LSM-710; Zeiss).

For ISG15 and PRV observation, PK15 cells were inoculated on the climbing plate. After treatment, 4% paraformaldehyde was fixed for 1 h, PBS was washed three times, and then directly incubated with anti-ISG15 (1: 200) and anti-PRV-gB (1: 500) antibody overnight at 4°C, followed by incubation with fluorescent secondary antibodies for 1 h at 25°C. Cell nuclei were stained by incubation with diamidino-2-phenylindole (DAPI) for 5 min, and observed under a confocal laser microscope (LSM-710; Zeiss).

### 2.6. Periodic acid-Schiff and Giemsa stain

For PAS stain, the sections are hydrated as previously mentioned and then treated with periodic acid for 7 min. They are rinsed under running water and soaked for 5 min. Next, the sections was soaked in Scheff’s reagent for 15 min in the dark and rinsed under running water. Then, the sections were counterstained with hematoxylin for 2 min and treated with acid differentiation solution for 3 s. Finally, the sections are subjected to conventional dehydration and transparency before being sealed.

After hydrating the sections as previously mentioned, apply a diluted Giemsa dye solution for 20 s (adjusting the dyeing time as needed), and rinse in water three times, for 2 minutes each time. Finally, the sections are subjected to conventional dehydration and transparency before being sealed.

### 2.7. TdT mediated dUTP Nick end labeling stain

Apoptosis was analyzed by TUNEL Cell Apoptosis Detection Kit (Alexa Fluor 488, Yisheng) which determines DNA fragmentation. The main steps are as follows: After sections hydration, incubate at room temperature for 20 min with Protease K, and moisten the sample with PBS solution for 2–3 times (positive control: treat with DNase I; negative control: replace TdT enzyme with ddH_2_O). Add 10 μL 1 × The equilibration buffer 25°C 20 min. Remove most of 1× Equilibration Buffer, then add 50 μL TdT incubation buffer. Keep out of light and incubate at 37°C for 60 min. The sections were placed in PBS solution and washed repeatedly for 3 times. Cell nuclei were stained by incubation with DAPI for 5 min, and observed under a confocal laser microscope (LSM-710; Zeiss).

### 2.8. Quantitative RT-PCR

Total RNA from nasal cavity mucosa and PK15 cells was extracted using a TRIzol reagent (Invitrogen) according to the manufacturer’s instructions. cDNA was generated by reverse transcription using HiScript™ Q RT SuperMix for qPCR (Vazyme, China) according to the manufacturer’s instructions. The target gene transcription was determined using quantitative RT-PCR (RT-qPCR) with the SYBR Green qPCR Kit (Takara, Beijing, China) and analyzed using the double standard curve method. All primers used for RT-qPCR are presented in Supplementary Table S1. Glyceraldehyde 3-phosphate dehydrogenase (GAPDH) was quantified as the internal control. The relative levels of cytokine RNA were calculated using the 2^-ΔΔ^ CT method.

### 2.9. Western blot analysis

Tissue and cell samples were assessed by western blotting with specific antibodies. At indicated times post-infection, the collected samples were lysed in RIPA buffer containing a protease inhibitor cocktail (Thermo Scientific). The concentration of the lysates was determined using a BCA protein quantification kit (Thermo Scientific). Equal amounts of protein were separated via SDS-PAGE and electrophoretically transferred onto a PVDF membrane (Millipore, Shanghai, China). The membranes were blocked with 5% skim milk for 1.5 h and incubated with anti-GAPDH (1:1,000), anti-caspase3 (1:1,000), anti-caspase9 (1:1,000), anti-PCNA (1:1,000) and anti-PRV-gB (1:3,000) overnight at 4°C, and followed by incubation with appropriate horseradish peroxidase-conjugated secondary antibodies. The intensity of the bands in terms of density was measured and normalized against GAPDH expression.

### 2.10. Plaque assay and ELISA analysis

Confluent monolayers of Vero E6 cells grown in 12-well tissue culture plates were infected with 500 μL of serial tenfold dilutions of the supernatant samples. After being incubated for 1 h at 37°C, cells were overlaid with 0.7% Sea-Plague agarose in DMEM containing 2% FBS and incubated at 37°C. Three days post-infection, the plaques were visualized via crystal violet staining.

The production of IFN-α, IFN-β and IFN-γ were detected using an ELISA kit (MEIMIAN), according to the manufacturer’s instructions.

### 2.11. NMEM were treated with PRV, sheep-derived *Bacillus subtilis*, *Streptococcus*, *Pasteurella multocida*, and lipopolysaccharide, respectively

The nasal mucosa explant was placed in 24-hole plate and inoculated with PRV (5 × 10^5^, 400 μL) to immerse the mucosal tissue. After 1 h, the nasal mucosal tissue was washed for 3 times and transferred to 12-hole transwell devices. The nasal mucosal was cultured at 37°C with 5% CO2. Infected nasal mucosa was collected at different time points (12, 24, 36, 48, 60 and 72 h) for subsequent analysis.

Th Sheep-derived *Bacillus subtilis*, *Streptococcus* and *Pasteurella multocida* were harvested by centrifugation at 300 g for 5 min, washed 3 times in PBS, and suspended to an OD600 of 0.6 (approximately 10^6^ CFU/ml) in PBS. Explants were washed with the cultivation medium without antibiotics and kept without antibiotics for at least 12 h prior inoculation. Next, explants were inoculated with 250 μL of bacterial suspension and allowed to adhere to the explants for 12 h at 37°C and 5% CO2. Drop the prepared bacterial solution with 250 μL onto the nasal mucosal explant cultured in the transwell devices, and allowed co-culture for 24 h. Then, the sheep nasal mucosa was collected for subsequent detection.

The LPS suspended to 0.01, 0.1, 1, 10 and 100 μg/ml in PBS. Explants were washed with the cultivation medium without antibiotics and kept without antibiotics for at least 12 h prior inoculation. Next, explants were inoculated with 250 μL of LPS of different concentrations and culture for 12 h at 37°C and 5% CO2, and wash the nasal mucosal tissue for 3 times. Then, the nasal mucosal culture it at 37°C with 5% CO2. The infected nasal mucosa was collected at 24 h for subsequent analysis.

### 2.12. Sheep-derived *Bacillus subtilis* and PRV were co-incubation with NMEM

Prior to the PRV infection, the sheep nasal mucosal explant model was pretreated with Sheep-derived *Bacillus subtilis* from the sheep nasal cavity, as outlined in Methods 2.11. The sheep nasal mucosa was moistened and washed three times with PBS. Following this, the PRV virus was inoculated according to Methods 2.11. After 24 h of culture, the collected samples were tested for viral load and distribution characteristics.

### 2.13. Intranasal administration with sheep-derived *Bacillus subtilis* on nasal mucosa

A total of eight newborn lambs were randomly divided into two groups of four each. The control group received an intranasal administration of PBS (500 μL per nostril), while the NSV2 group received intranasal administration of 109 CFU/ml at 12, 21, and 26 days after birth. The dosage for each administration was 0.5 ml, 1 ml, and 1 ml, respectively. At 30 days, lambs were euthanized using intravenous injection of sodium pentobarbital (100 mg/kg). The nasal cavity was then opened and 5 ml of PBS was added to rinse the cavity. The rinse solution was collected from the nostrils and the content of IFN-α/β/γ was detected using ELISA. Additionally, fixed and molecular samples of the nasal mucosa were collected to detect the expression of ISG15.

### 2.14. Sequencing and isolation of sheep-derived nasal flora

The nasal swabs collected from the stocking (*n* = 4) and feeding (*n* = 4) sheep, respectively. After the sheep are pinioned, the sterile swab inserts the nasal cavity for more than 10 cm and rotate it for 3 times. DNA was extracted from nasal swabs using HiPure soil DNA kit (Magen). 16S ribosomal RNA (rRNA) V3-V4 was amplified with specific primers (F: 5’-GTGCCAGCCGGGTAA-3′; R: 5′- GGACTACHVGGGTWTCTAAT-3′). The HiSeq 2500 system (Illumina Inc) was used used for sequencing and the USEARCH (v7.0.1090) is used to cluster the sequences into operation classification units (OTUs), which have 97% identity. The relative abundance of OTUs and classify was calculate at different taxonomic levels (phyla, order, class, family, genus and species). Based on OTU and species annotation, the complexity of sample species and species differences among populations were analyzed.

In the meantime, the centrifuge tube containing the nasal swab was open in the ultra-clean table aseptically, and add 500 μL of PBS solution, and then shake repeatedly three times. The leachate was collected and heat in 80°C water bath for 15 min, and then transfer the 100 μL leaching solution is transferred to LB (Invitrogen) and cultured overnight at 37°C and 220 rpm. According to the colony morphology and Gram staining results select single colony. After colony suspension was prepared, 16S rDNA universal primers 27F and 1492R were used primers for PCR amplification. Primer sequence 27F: AGAGTTTGATGCTCAG, 1492R: TACCTTTGTTACGACTT. The PCR product was sent to Shanghai Shenggong Biotechnology Co., Ltd. for sequencing, and was compared by NCBI blast. According to the sequence comparison results, all strains of *Bacillus subtilis* group were stored for the next test.

### 2.15. RNA interference of ISG15

The PK15 were transfected with 100 nM ISG15-specific or scrambled control siRNA duplexes (Supplementary Table S2) using the X-tremeGENE HP DNA transfection reagent (Roche) according to the manufacturer’s instructions. After 36 h of transfection, the cells were inoculated with 250 μL of bacterial suspension for 12 h, infected with PRV (MOI = 0.1) 12 h for subsequent analysis.

### 2.16. Data analysis

Results are presented as means ± SD and analyzed with SPSS 17.0. One-way ANOVA was employed to determine statistical differences among multiple groups, and T-test was employed to determine differences between the two groups. *: *p* < 0.05. Different letters also indicate significant differences.

## 3. Results

### 3.1. Establishment of a sheep NMEM

The integrity and vitality of the NMEM are crucial for further experimental research and practical applications. To ensure this, we have evaluated the activity of the NMEM through multiple indicators. Firstly, we observed that the cilia of sheep nasal mucosa explants vibrated at a high speed for about 11 days (Figure 1B; Supplementary Video). Furthermore, the integrity of the nasal mucosal explant was good, and the expression of cytokeratin 18 and the tight junction protein claudin-1 in the epithelial layer of the explant was high. PAS staining was used to observe the secretory characteristics of the sheep nasal mucosa explants, and the secretory capacity of the gland decreased with longer culture time (Figure 1C). At last, CD3^+^ T-, IgA^+^ B-, and CD208^+^-positive cells (antigen markers of sheep dendritic cells) (Salaun et al., 2004) were distributed in the sheep nasal mucosa explant of 6-month-old sheep (Supplementary Figure S1).

**Figure 2:**
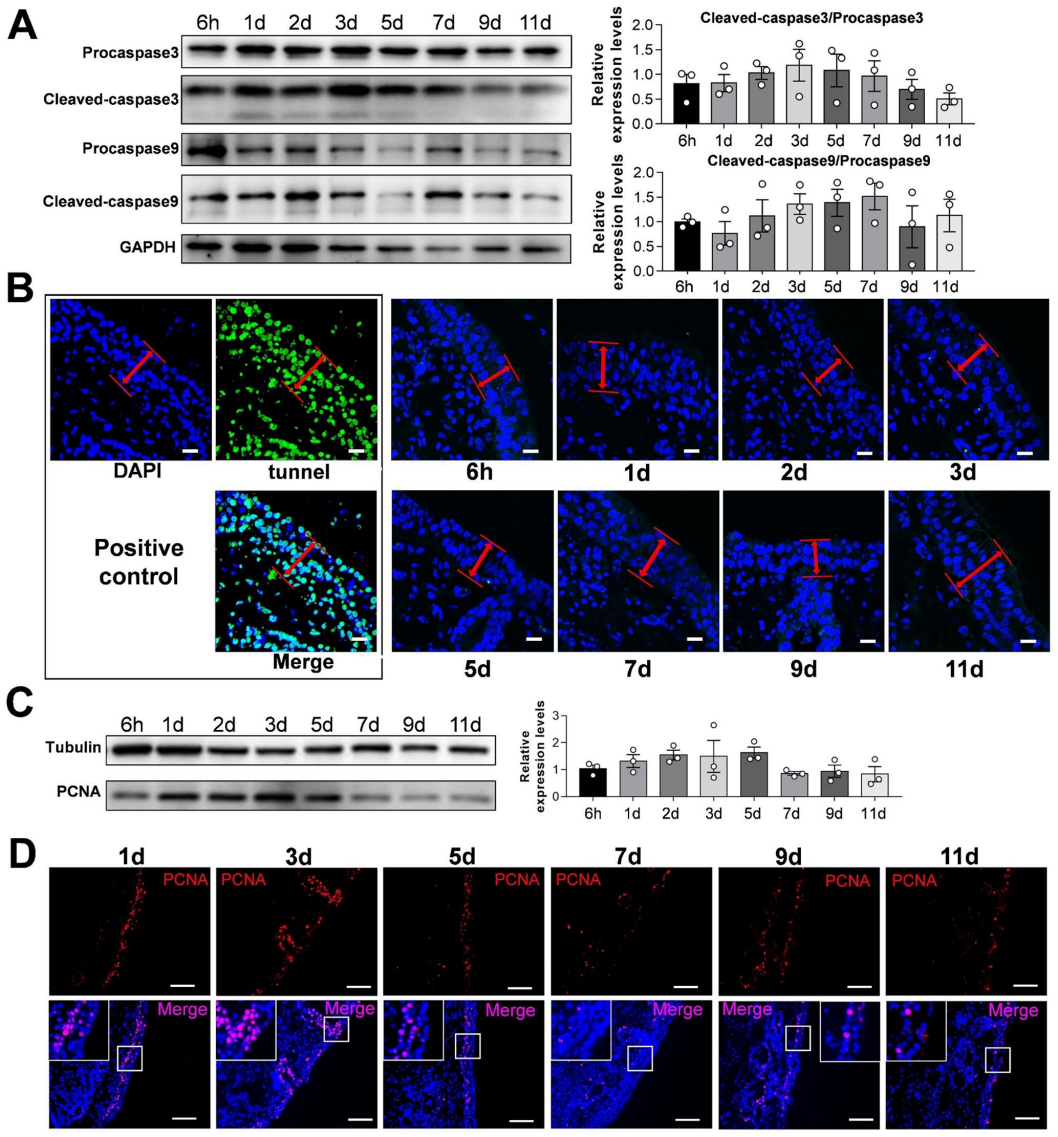
Detection of apoptosis and proliferation of sheep nasal mucosa explants. **(A)** Western blot was used to detect the expression of Procapase3, Cleaved-caspase3, Procapase9 and Cleaved-caspase9 in sheep nasal mucosa explants at different culture time points (6 h, 1 d, 2 d, 3 d, 5 d, 7 d, 9 d, and 11 d). Statistical diagram of protein expression of Cleaved-caspase3/Procapase3 and Cleaved-caspase9/Procapase9. **(B)** Tunnel kit was used to detect the apoptosis of sheep nasal mucosa explants at different time points (6 h, 1 d, 2 d, 3 d, 5 d, 7 d, 9 d, and 11 d). The red arrows represent the epithelial layer **(C,D)** Western blot and immunofluorescence were used to detect the proliferation characteristics of nasal mucosa explants at different time points. The nasal mucosa explants of sheep at different times were obtained from three mucosal tissues of three sheep. Scar bar = 50 μm.

To verify the reliability of the culture model, we detected apoptosis and proliferation in the cultured sheep nasal mucosa explants. The results indicated no significant differences between the ratios of cleaved-caspase-3/procapase-3 and cleaved-caspase-9/procapase-9 with increased incubation time (Figures 2A,B). TUNEL assay revealed no apoptosis after 1–11 days compared with the positive control group (Figure 2C). Few apoptotic cells were observed under the epithelial layer of the nasal mucosa (Supplementary Figure S3). The nasal mucosa explants exhibited good proliferative ability within the first 3 days, which decreased gradually thereafter (Figures 2C,D). Furthermore, the expression levels of innate immunity factors decreased as the culture time of nasal mucosal explants increased (Supplementary Figure S2A). The expression pattern of innate immune factors at different times closely resembled that observed *in vivo* (Supplementary Figure S2B).

### 3.2. Establishment of infection model of sheep nasal mucosa explants

**Figure 3:**
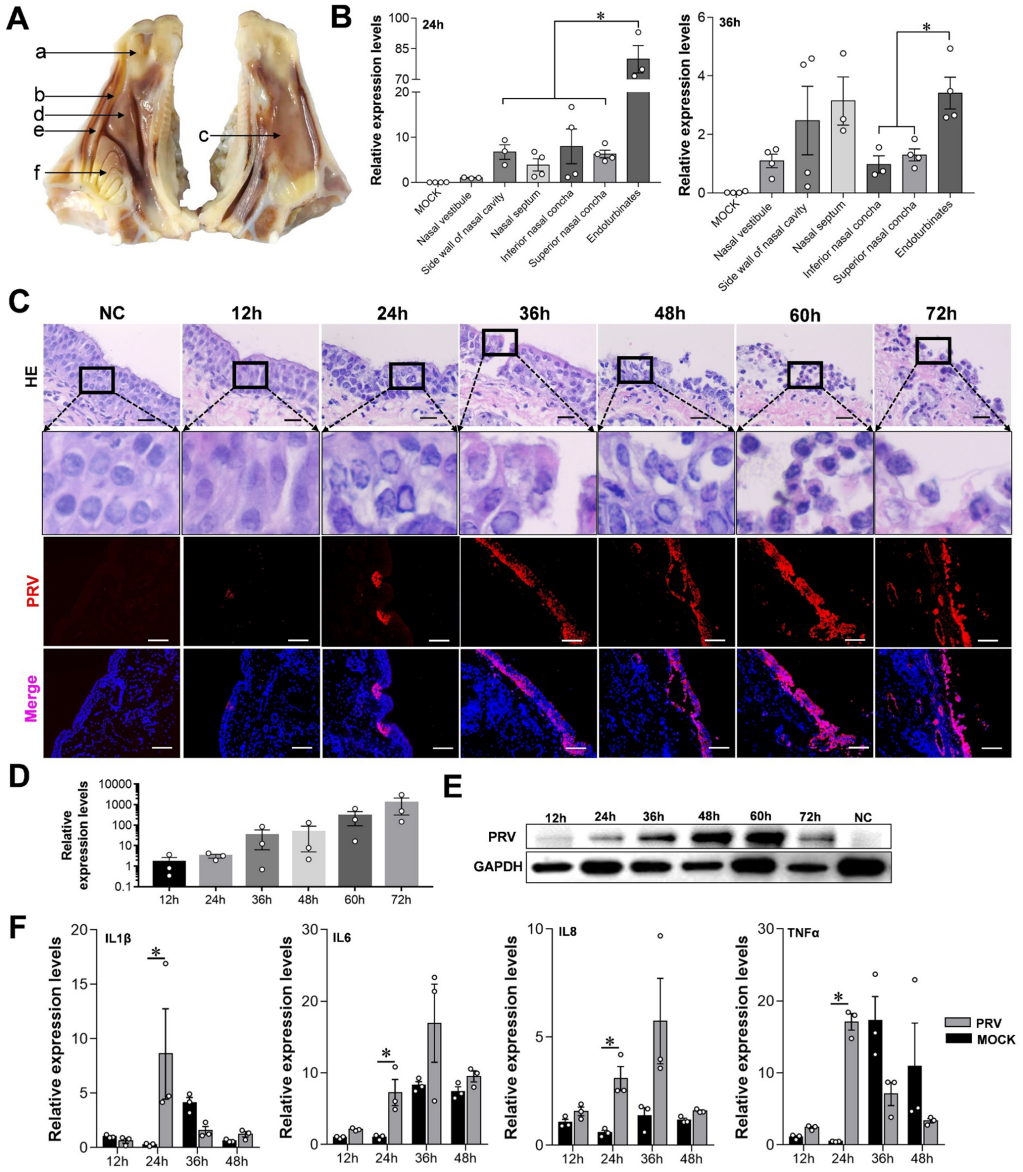
Characteristics of sheep nasal mucosal explant infected with PRV. **(A,B)** Quantitative detection of PRV infection characteristics in different parts of sheep nasal mucosa explants. **(A)** Nasal vestibule, **(B)** side wall of nasal cavity, **(C)** nasal septum, **(D)** inferior nasal concha, **(E)** superior nasal concha, **(F)** endoturbinates. **(C)** HE staining and immunofluorescence for evaluate the pathological characteristics of sheep nasal mucosa explants infected with PRV at different times (12, 24, 36, 48, 60, and 72 h). **(D,E)** Quantitative detection of PRV infection characteristics at different times (12, 24, 36, 48, 60, and 72 h). **(F)** The affection of PRV infected sheep nasal mucosa explants on innate immune factors. The nasal mucosa explants of sheep were obtained from three mucosal tissues of three sheep. Scar bar = 100 μm.

PRV can cause fatal infection in sheep, and even attenuated vaccines may cause an outbreak of Pseudorabies in sheep (Kong et al., 2013). Therefore, we constructed a model of PRV-infected sheep nasal mucosa explants to explore the infection and characteristics of PRV. The results show that the endoturbinates explants, located in the olfactory region of the sheep’s nasal cavity, had a significantly higher PRV infection level at 24 and 36 h compared to other tissues such as the nasal vestibule, inferior and superior nasal concha (Figures 3A,B). As the infection time increased, the nuclei of the mucosal epithelium became vacuolated after 24 h, the upper cortex detached after 48 h, and almost all epithelial layers detached at 72 h (Figure 3C). The results of immunofluorescence and western blot assays were consistent, and the PRV viral load increased significantly over time (Figures 3D,E). Additionally, the expression of the inflammatory factors interleukin (IL)-1β, IL-6, IL-8, and tumor necrosis factor (*TNF*)-*c* in the PRV infected group was higher than in the control. However, the expression of inflammatory factors increased at other time points, although not significantly, and some inflammatory factors were downregulated (Figure 3F).

*Streptococcus* and *Pasteurella multocida* are important pathogenic microorganisms colonized in sheep nasal cavities (Scott, 2011; Kumar et al., 2014). In this study, HE staining results showed that the cilia integrity of mucosal explants treated with *Streptococcus* and *Pasteurella multocida* became worse compared with the control group (Supplementary Figure S4B). Besides, we observed the presence of Pasteurella-like bacteria in the sheep nasal mucosa through Giemsa staining (Supplementary Figure S4B). When the nasal mucosa explants of sheep were pretreated with *Streptococcus* and *Pasteurella multocida*, the expression levels of inflammatory factors such as *IL-1β*, *IL-6*, *IL-8*, and *TNF-α* were significantly increased (Supplementary Figure S4C). Lipopolysaccharide (LPS) is a component of the outer wall of the cell wall of gram-negative bacteria, which can effectively induce host inflammation. After being treated with LPS, the expression levels of inflammation-related factors, including *IL-1*, *IL-6*, and *TNF-α* were significantly increased in sheep nasal mucosa explants. These results were consistent with those of pathogen treatment, and have a significant concentration dependent (Supplementary Figures S5A,B).

### 3.3. Isolation and identification of sheep nasal cavity flora

In addition to pathogenic bacteria and viruses, there are also a large number of commensal microorganisms in the nasal cavity, which is essential to maintain the health and stability of the respiratory tract (Bosch et al., 2013). A 16sRNA sequencing system was used to compare the structural characteristics and differences in nasal flora between stocking and feeding sheep. There is no significant difference in diversity and characteristics of flora between Stocking and Feeding (Supplementary Figure S4A). Based on the permutational multivariate analysis of variance and the principal coordinates analysis (PCoA), there was significant difference in nasal microbes between Stocking and Feeding (Supplementary Figure S4B). There are 1,386 unique OTUs in Stocking, 1,455 unique OTUs in Feeding group (Supplementary Figure S4C). Stocking and feeding sheep are mainly enriched in Firmicutes (17.7%/38.0%), Proteobacteria (17.2%/28.3%), Tenericutes (14.2%/10.5%), Actinobacteria (20.1%/14.5%), and Bacteroidetes (12.5%/4.0%) (Supplementary Figure S4D). Statistics were made on the top 35 genera with the highest enrichment, mainly including *Staphylococcus* (15.7%/78%), *Mycoplasma* (1.5%/12.0%), *Moraxella* (3.0%/11.9%), *Filamentous bacteria* (12.4%/1.5%) and *Acinetobacter* (1.1%/6.0%) (Supplementary Figure S4E). The abundance of Bacillus spp. in the stocking group was significantly higher than in the Feeding group (*p* < 0.05, Supplementary Figure S4F).

Accordingly, we isolated Bacillus strains from the nasal cavities of sheep and observed that their colony morphology matched the Gram staining characteristics of *Bacillus subtilis* group (Supplementary Figures S4G,H). Furthermore, we sequenced the *Bacillus* in the sheep nasal cavity and obtained six *Bacillus subtilis* strains (NSV1, NSV2, NSV3, NSS1, NSS2, and NSP1) and three unclassified Bacillus strains (NSS3, NSS4, and NSS5) (Supplementary Figure S4F).

### 3.4. Sheep-derived *Bacillus subtilis* enhances the innate immunity of sheep nasal mucosa explants

**Figure 4:**
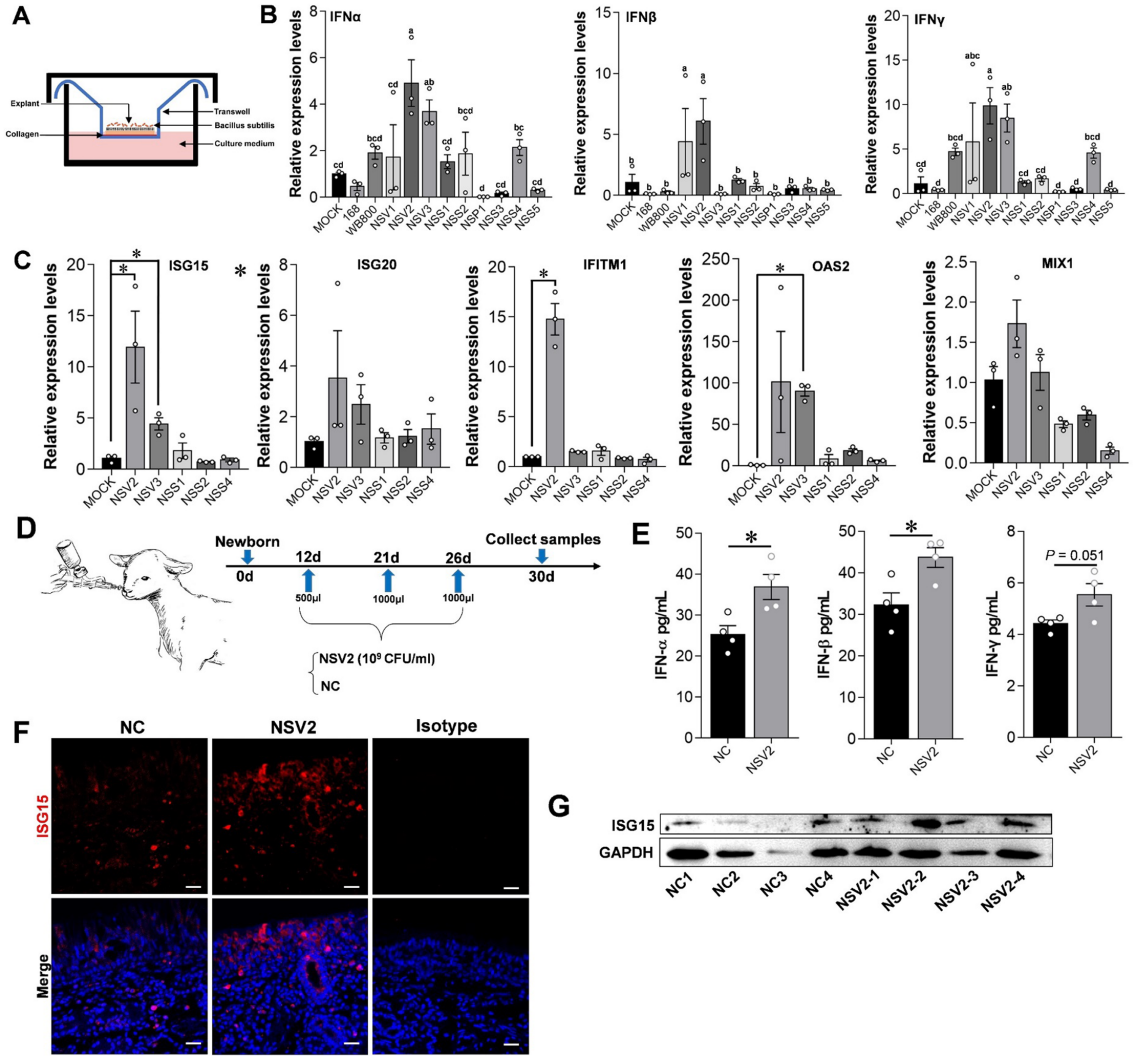
The effect of sheep-derived *Bacillus subtilis* on innate immune factors in nasal cavity. **(A,B)** Quantitative detection of *IFN-α*, *IFN-β*, and *IFN-γ* in sheep nasal mucosa explants with pretreat sheep-derived *Bacillus subtilis* (NSV1, NSV2, NSV3, NSS1, NSS2, NSP1, NSS3, NSS4, and NSS5). **(C)** Quantitative detection of *ISG15*, *ISG20*, *IFITM1*, *OAS2*, and *MIX1* in sheep nasal mucosa explants with pretreat sheep-derived *Bacillus subtilis* (NSV2, NSV3, NSS1, NSS2, and NSS4). The nasal mucosa explants of sheep were obtained from three mucosal tissues of three sheep. **(D)** The sheep derived NSV2 was used to pray the nasal cavity of the lamb (*n* = 4) three times. **(E)** The levels of IFN-α, IFN-β and IFN-γ in nasal lavage fluid after treatment with NSV2 detected by ELISA. **(F,G)** The expression of ISG15 detected by immunofluorescence and Western blot. Different letters indicate significant differences. Scar bar = 20 μm.

The results showed that treating sheep nasal mucosa explants with sheep-derived *Bacillus subtilis* strains NSV2 and NSV3 significantly increases the expression of *IFN-α*, *IFN-β*, and *IFN-γ* (*p* < 0.05). NSV1, NSS2, and NSS4 tended to upregulate IFNs expression (Figures 4A,B). Then, we detected the ISGs downstream of interferon. NSV2 and NSV3 can significantly induce the expression of ISG15, and significantly upregulate IFITM1 and OAS2, respectively (Figure 4C). Furthermore, NSV2 nasal spray lamb significantly induced IFN-α and IFN-β (Figures 4D,E). The immunofluorescence and Western blot results showed that the expression of ISG15 increased significantly in the nasal mucosa (Figures 4F,G).

### 3.5. Sheep-derived *Bacillus subtilis* from nasal cavity inhibits PRV infection of nasal mucosa explant

**Figure 5:**
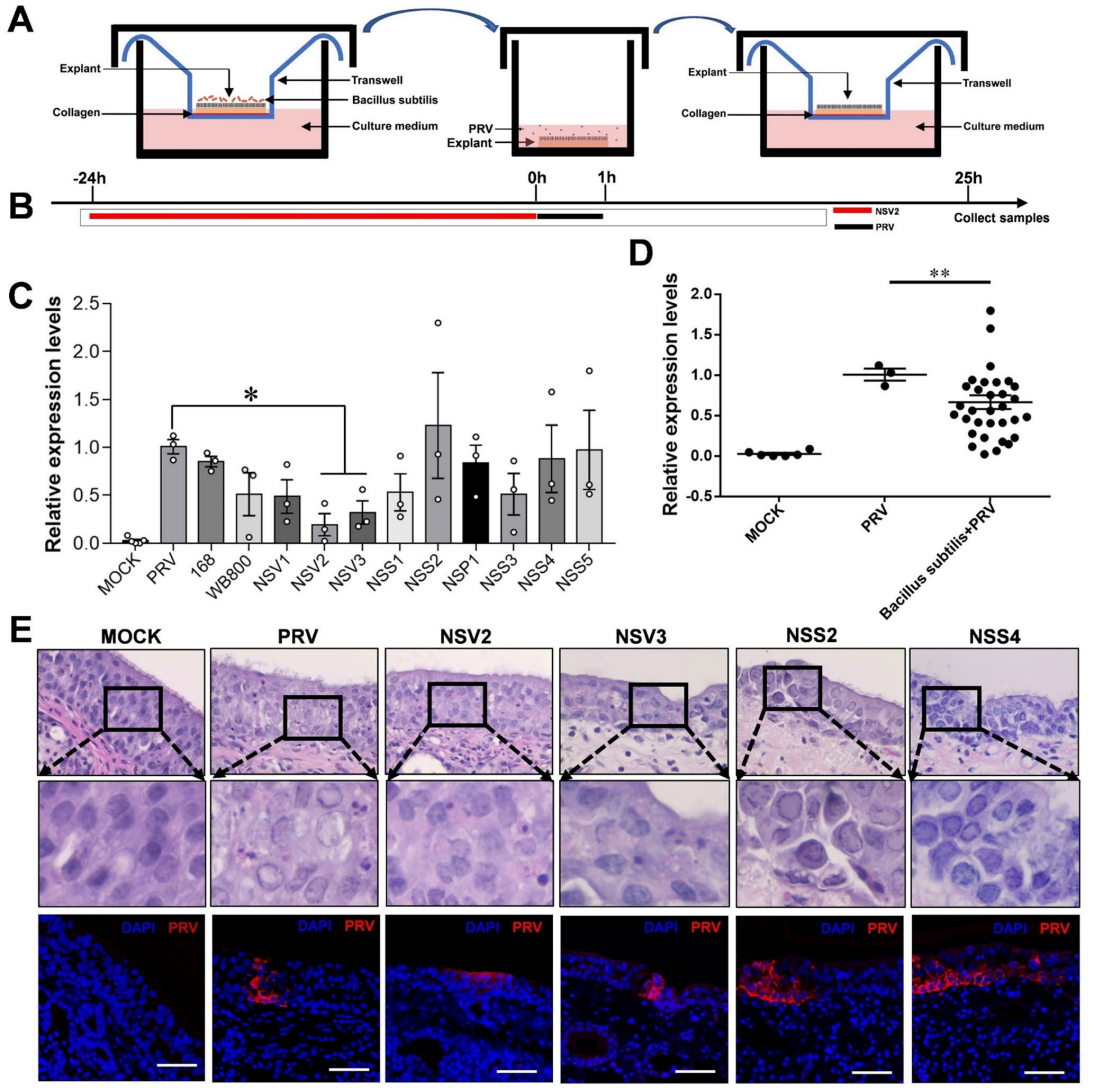
The Inhibitory effect of sheep-derived *Bacillus subtilis* on PRV in nasal mucosa explants. **(A,B)** After the mucosal explants were pretreated by *Bacillus subtilis* for 24 h, they were transferred to a 24 well plate, inoculated with PRV for 1 h, and then transferred back to the transwell devices to culture for 24 h to collect samples. **(C,D)** Quantitative detection of PRV viral load after treatment with different sheep-derived Bacillus strains. **(E)** HE staining and immunofluorescence for evaluate the pathological characteristics after treatment with different sheep-derived Bacillus strains. The nasal mucosa explants of sheep were obtained from three mucosal tissues of three sheep. Scar bar = 50 μm.

In order to further explore the possible interaction of sheep nasal microorganisms. We used the nasal mucosal explants model pretreated with sheep-derived *Bacillus subtilis* of different strains (Figures 5A,B). The result showed that NSV2 and NSV3 significantly inhibited PRV infection of the sheep nasal mucosal explants (Figures 5C,D). Hematoxylin and eosin (HE) staining and immunofluorescence observations revealed that the degree of pathological change and fluorescence intensity of PRV in the NSV2 and NSV3 treatment groups were significantly lower than those in the control group (Figure 5E).

### 3.6. Sheep-derived *Bacillus subtilis* resists PRV infection by inducing ISG15 expression

**Figure 6:**
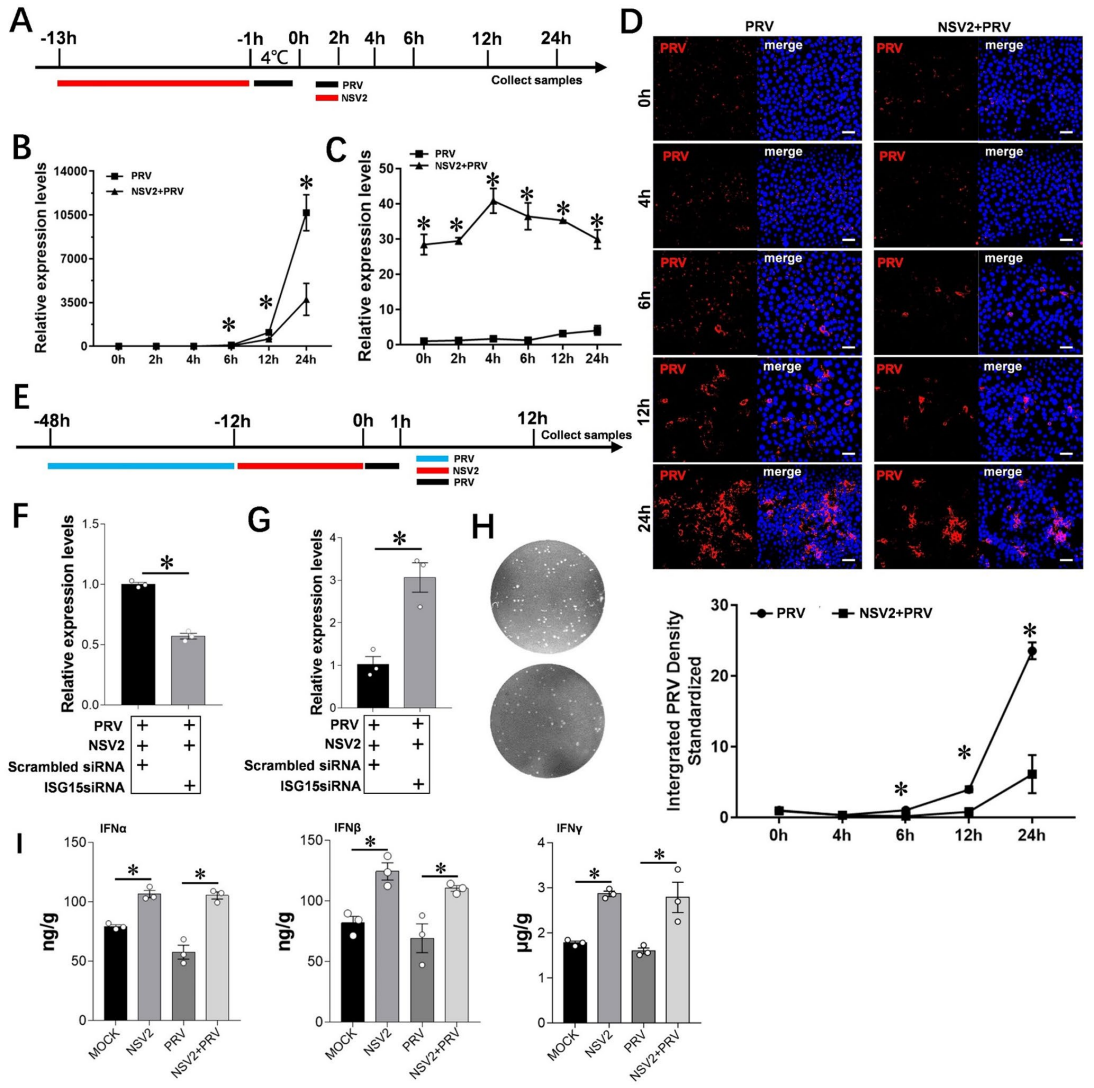
Interference with ISG15 to inhibit the resistance of NSV2 to PRV infection. **(A)** After the PK15 were pretreated by NSV2 for 12 h, inoculated with PRV (MOI = 0.1) for 1 h, and then collect samples at different time points (0 h, 2 h, 4 h, 6 h, 12 h and 24 h). **(B** and **C)** Quantitative detection of PRV and ISG15 levels after treatment with NSV2 at different time points. **(D)** The pathological characteristics after treatment with NSV2 detected by immunofluorescence at different time points. **(E)** After 36 h of transfection with siISG15, the cells were inoculated with 250 μL of NSV2 suspension for 12 h and infected with PRV (MOI = 0.1) for 1 h and then collect samples at 12 h. **(F–H)** Quantitative detection of ISG15 and PRV levels, and detection the viral load in culture supernatants with plaque assay. **(I)** The levels of IFN-α, IFN-β and IFN-γ after treatment with NSV2 detected by ELISA. Scar bar = 50 μm.

To further explore the role of Sheep-derived *Bacillus subtilis* in resisting PRV infection, we selected PK15 as a cell model to explore its specific mechanism. qPCR results showed that the level of viral mRNA in cells treated with *Bacillus subtilis* was significantly lower than that in control cells (Figures 6A,B). The results of immunofluorescence assay showed that the PRV fluorescence intensity at 6, 12, and 24 h in the *Bacillus subtilis* treatment group was significantly lower than that of the control group (Figure 6D). ISG15 expression was high within the first 24 h after *Bacillus subtilis* treatment (Figure 6C). In order to further explore the role of ISG15 in the inhibition of pseudorabies virus by *Bacillus subtilis*, we designed the interfering RNA of ISG15 for further exploration (Figure 6E). The results showed that after using siISG15, the ability of *Bacillus subtilis* to inhibit PRV infection decreased significantly compared with Scrambled RNA (Figures 6F–H), and the content of upstream IFN-α/β/γ significantly increased (Figure 6I).

### 3.7. Sheep-derived *Bacillus subtilis* inhibits PRV infection through ISGlation

**Figure 7:**
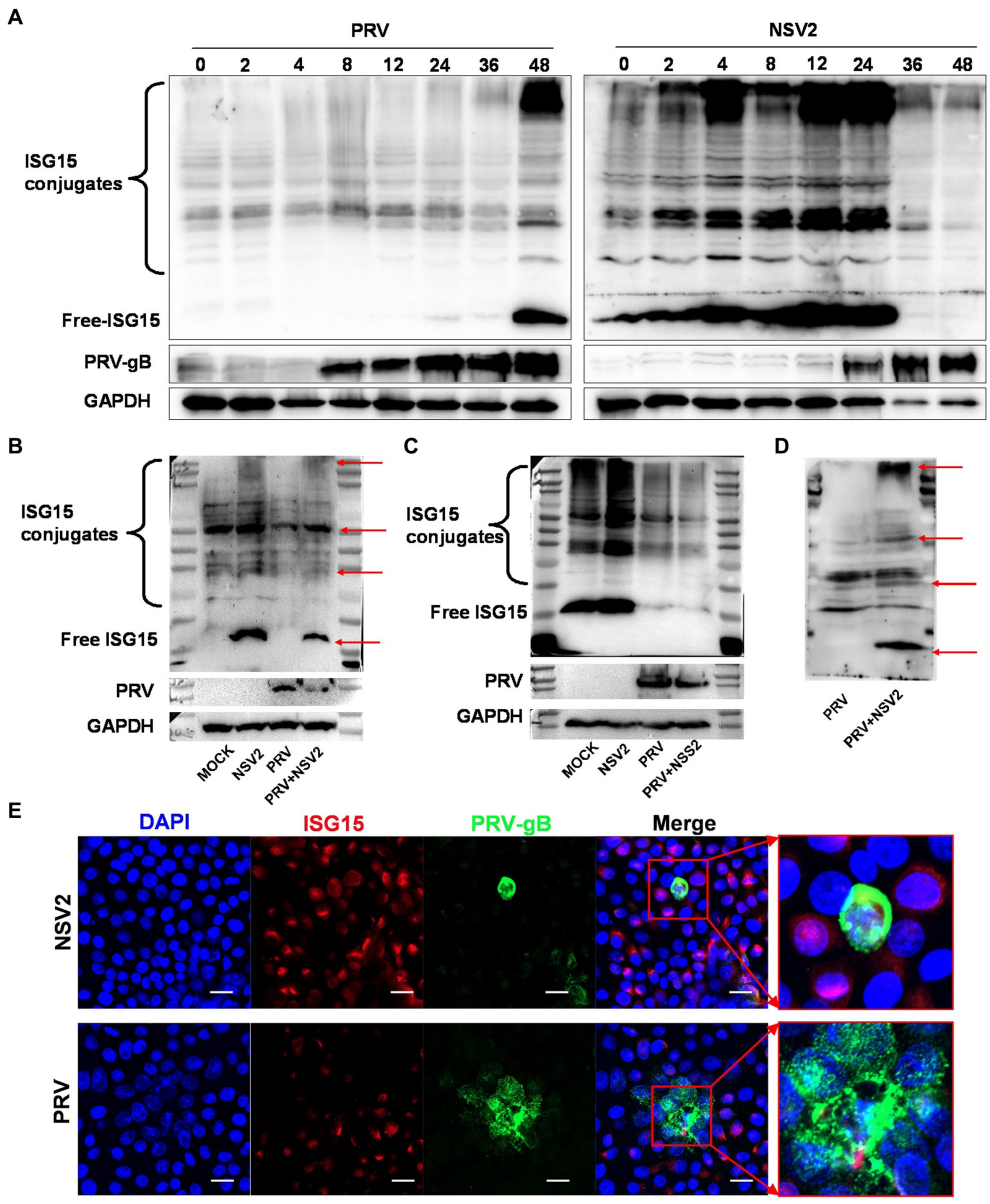
NSV2 inhibit PRV infection by ISGylation. **(A)** The expression and binding of ISG15 in PK15 cells infected with PRV and treated with NS2 at different time points were detected by Western blot. **(B–D)** Comparison of ISG15 expression and binding between PK15 cells infected with PRV and treated with NSV2/NSS1/NSV3. **(E)** The colocalization of PRV and ISG15 detected by immunofluorescence. Scar bar = 20 μm.

ISG15 is an important ubiquitin-like protein that significantly inhibits viral replication by binding to host or viral proteins. Samples with a parallel infection in the *B. subtilis* treatment group were analyzed by western blot, to confirm the results and determine the ISG15 ubiquitination level after treatment with *B. subtilis*. The PRV gB protein was first detected at 0 h (Figure 7A) and continued to increase until 48 h. The results showed that the accumulation of free ISG15 was time-dependent and became obvious at 36 h, while the increase of ISG15 conjugate was not obvious until 36 h. Compared with the accumulation of PRV-gB protein, the increase of ISG15 protein has a 24-h delay (Figure 7A). However, significant differences can be seen in the treatment group of *Bacillus subtilis* NSV2. After NSV2 treatment, a large amount of free ISG15 was accumulated in the early stage of PRV infection, while a large number of ISG15 conjugates appeared in the early stage of infection (Figure 7A). At the same time, the expression of PRV-gB protein also appeared in large numbers after 24 h (Figure 7B). To further compare the presence of free and bound ISG15 after treatment with *Bacillus subtilis*, we detected the blank group, PRV group, *Bacillus subtilis* group and *Bacillus subtilis* + PRV group 12 h after virus infection. The results showed that NSV2 could significantly enhance the expression of free ISG15 and the accumulation of bound ISG15 (Figure 7B). In addition, the results were consistent with that of ISG15 induced by *Bacillus subtilis* in explants, the NSS2 could not induce its expression (Figure 7C) and NSV3 could significantly enhance the expression of ISG15 (Figure 7D). Morever, immunofluorescence results indicate a significant co-localization between ISG15 and PRV-gB (Figure 7E).

## 4. Discussion

The nasal cavity is the primary target for respiratory tract infection by pathogenic microorganisms. Nasal mucosa explants, which retain normal cell–cell contact and consequently maintain the three-dimensional structure of the tissue, have been used in pathogenic research (Glorieux et al., 2007; Abeynaike et al., 2010; Van Poucke et al., 2010; Alfi et al., 2020). Human nasal mucosal explants were cultured on a strip of filter paper, with the two ends immersed in MEM for up to 20 d and the medium replaced daily; only the ciliary beat frequency was detected (Jackson et al., 1996). The porcine nasal mucosa has been cultured in an air-liquid interface for up to 72 h (Van Poucke et al., 2010; Tulinski et al., 2013). Furthermore, sheep nasal mucosa explants were cultured in an air-liquid interface system in 6-well plates for 5 d (Di Teodoro et al., 2018; Mazzetto et al., 2020). In this study, we established an air-liquid culture model of the sheep nasal mucosa at the air-liquid interface for up to 11 d. On day 11, we observed high-speed oscillations of the cilia, with tight junction proteins and high proliferative activity. Previous studies have trimmed the implant to a 50-mm^2^ (Mazzetto et al., 2020) or 16-mm^2^ (Glorieux et al., 2007) square to ensuring consistency of the size of mucosal explants. However, the size of mucosal explants can be controlled using an 8-mm-diameter tissue sampler, which provides consistent results over repeated tests.

Antiviral research is typically conducted through experiments using cell and animal models. However, *in vitro* experiments overlook the physiological differences between *in vitro* and *in vivo* experiments, and *in vivo* experiments are costly. Explant model systems are highly valuable tools as they maintain the tissue cytoarchitecture, allowing for a more accurate representation of what occurs at the organismal level (Grivel and Margolis, 2009). Consequently, they serve as a useful steppingstone for bridging the gap between *in vitro* and *in vivo* drug evaluations, including antiviral drugs. This suggests that the NMEM is a dependable model for investigating antiviral effects. Additionally, utilizing discarded material is a cost-effective alternative to live animal testing and aligns with the 3R concept, which aims to reduce the number of experimental animals required (Diaz et al., 2020). In fact, past research has employed explants as antiviral models and yielded promising outcomes (Pennington et al., 2016; Putra et al., 2018). This indicates that the NMEM is a reliable model for studying antiviral effects. PRV is a multi-host infection virus that can reproduce in epithelial cells of the nasal and oral mucosa, and transmit through peripheral nerves to cause severe infections (Yang et al., 2012; Gu et al., 2015). A previous study used PRV NIA-3 to infect a pig nasal mucosal explant, and IFN-α1 treatment significantly inhibited infection (Pol et al., 1991); it has also been used to compare the infection characteristics of seven European PRV strains in pig nasal mucosa (Glorieux et al., 2009). Another study demonstrated that US3 plays an important role in PRV invasion of the mucosa across the basement membrane through the nasal mucosal explant system (Lamote et al., 2016). Although previous studies have examined the activity of explant culture models and virus infection characteristics, they have not done so in a systematic manner. Therefore, the purpose of this study was to conduct a thorough evaluation of PRV-infected NMEM, which will serve as a basis for future antiviral drug screening and investigation into the role of nasal symbiotic microorganisms in resisting viral infections.

Studies have demonstrated that the microbiota in the mucosal layer is key for restricting the invasion and infection of various pathogens (Bassis et al., 2014; Man et al., 2017; Spacova et al., 2021). The composition and types of respiratory microorganisms affect the innate immune response, which plays an important role in the health of the host (Pettigrew et al., 2012; Thaiss et al., 2016; Brown et al., 2017; Morton and Singanayagam, 2022). A previous study isolated 1,556 bacteria were isolated from 960 nasal swabs collected from three different highland areas of Ethiopia. The total isolation rate of *Bacillus* spp. was 10.6% (3rd), and the isolation rate in cases with no clinical signs was higher than that of cases with respiratory clinical signs (Tesfaye et al., 2013). *Bacillus* has also been isolated from the nasal cavity of wild and domestic sheep (Queen et al., 1994). In another study, the isolation rate of *Bacillus spp.* from the nasal cavity of wild sheep was 22.4% (Marshall et al., 1983). In this study, the abundance of *Bacillus spp.* (mainly the *Bacillus subtilis* group) in the nasal cavity of the stocked sheep was significantly higher than in the feeding sheep. The number of immune cells and expression of innate immune factors in the nasal cavity of pigs increases significantly with the use of a *Bacillus subtilis* nasal spray (Yang et al., 2018). *Bacillus velezensis* isolated from yaks enhances antioxidant activity, the expression of cytokines related to immunity, and inflammation in mice (Li et al., 2019). In the present study, sheep-derived *Bacillus subtilis* significantly induced the expression of innate immune factors in the nasal mucosa explants. Similar results were observed in an *in vivo* nasal spray test, which further demonstrated that the sheep NMEM is a reliable *in vitro* model for simulating *in vivo* conditions.

The natural antiviral immunity in the respiratory tract is mediated by increased secretion of IFNs (Onoguchi et al., 2007; Ye et al., 2019; Sposito et al., 2021). Nasal commensal *Staphylococcus epidermidis* enhances IFN-λ dependent immunity against the influenza virus (Kim et al., 2019). In this study, to further explore the potential role of *Bacillus subtilis* in the nasal cavity, we used sheep nasal cavity mucosal explants pretreated with NSV2, which significantly inhibited PRV infection. The levels of ISG15 in the sheep nasal mucosa explant and PK15 cells treated with NSV2 consistently remained high. ISG15 is a ubiquitin-like protein that is induced by type-I IFN and functions as a protein regulator with multifunctional effects (Perng and Lenschow, 2018). Overexpression of ISG15 inhibits PRV replication (Liu et al., 2018), but the specific mechanism is unclear. ISG15 directly inhibited virus infection through ubiquitin-like viral proteins (Perng and Lenschow, 2018). Studies on influenza viruses show that the ubiquitination of NS1/A directly limits its ability to destroy the antiviral response of the host and inhibit its replication (Zhao et al., 2016). Moreover, ISG can be used as an immune regulatory protein to regulate the host immune response during viral infection and indirectly inhibit viral infection. IRF3 is ubiquitinated during infection with the Sendai virus, which inhibits the interaction between IRF3 and peptidyl-prolyl cis-trans-isomerase NIMA interacting 1, thereby preventing the ubiquitination and subsequent degradation of IRF3 (Shi et al., 2010) and enhancing the host’s immune response. In this study, on comparing the results of the different treatment groups, we found that ISGs was upregulated by *Bacillus subtilis*, possibly via enhanced ubiquitination of the host protein, inhibition of ubiquitination, and degradation of the host cell protein by PRV, thus inhibiting PRV infection.

**Figure 8:**
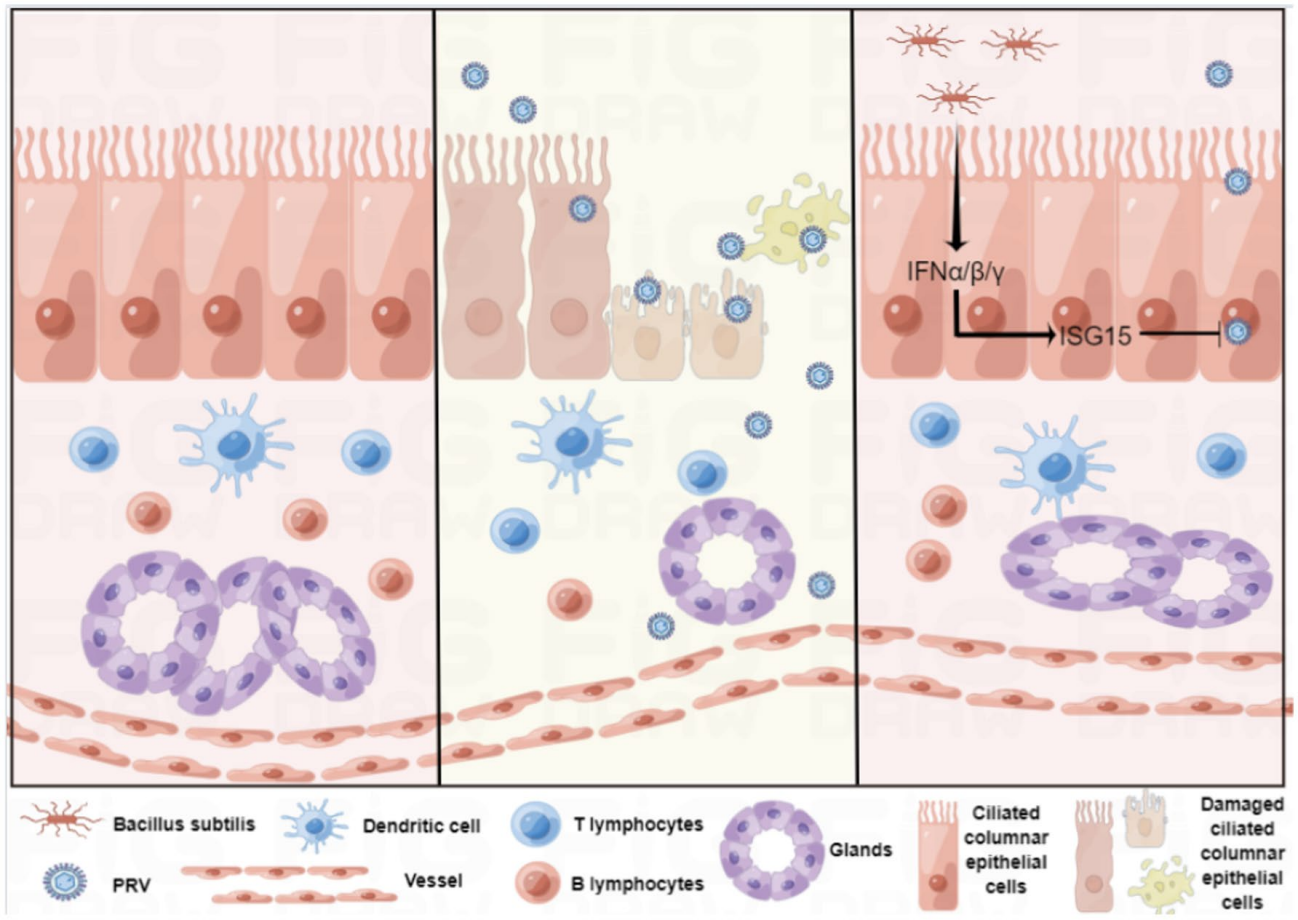
Schematic of the establishment of the sheep nasal mucosa explant model and its application.

In conclusion, we established a reliable *in vitro* culture model of sheep nasal mucosal explant, and applied it in antiviral research (Figure 8). sheep nasal mucosa was cultured in vitro for an extended period, and good activity and proliferative ability were observed. After treatment with by PRV, the sheep nasal mucosa caused a significant pathological change and mucosal immune response. Furthermore, nasal symbiotic NSV2 can significantly resist PRV infection by inducing ISG15 in sheep nasal mucosal explant.

## Data availability statement

The data presented in this study are deposited in the NCBI repository, accession number PRJNA970678.

## Ethics statement

The animal study was reviewed and approved by the Institutional Animal Care and Use Committee of Nanjing Agriculture University and followed National Institutes of Health guidelines for the performance of animal experiments. Written informed consent was obtained from the owners for the participation of their animals in this study.

## Author contributions

JZ was responsible for performing the experiments, analyzed the data, and prepared the manuscript. JL designed and carried out the molecular genetic studies, and participated in the preparation and revision of the manuscript. YM, CY, QZ, and YL were responsible for data verification and manuscript polish. QY conceived and designed the study and revised the manuscript. All authors contributed to the article and approved the submitted version.

## Funding

This work was supported by the National Natural Science Foundation of China (32072835 and 31930109), the Project of Sanya Yazhou Bay Science and Technology City (Grant No: SCKJ-JYRC-2022-31) and the Natural Science Foundation of Jiangsu Province (BK20190077) to JL, a Project Funded by the Priority Academic Program Development of Jiangsu Higher Education Institutions (PAPD) to QY, and Natural Science Foundation of Jiangsu Province grant (BK20200536), the State Key Laboratory of Veterinary Etiological Biology, Lanzhou Veterinary Research Institute, Chinese Academy of Agricultural Sciences (SKLVEB2021KFKT001) to YL.

## Conflict of interest

The authors declare that the research was conducted in the absence of any commercial or financial relationships that could be construed as a potential conflict of interest.

## Publisher’s note

All claims expressed in this article are solely those of the authors and do not necessarily represent those of their affiliated organizations, or those of the publisher, the editors and the reviewers. Any product that may be evaluated in this article, or claim that may be made by its manufacturer, is not guaranteed or endorsed by the publisher.
