# Supplementary material for: Establishment of sheep nasal mucosa explant model and its application in antiviral research
Source: Front Microbiol. 2023 May 15;14:1124936. doi: 10.3389/fmicb.2023.1124936 (PMC10226428; doi:10.3389/fmicb.2023.1124936)
Supplement: Supplementary file 1 [file Data_Sheet_1.docx]

Table S1 Primers used for real-time PCR

| **Gene** | **Forward** | **Reverse** | **Species** |
| --- | --- | --- | --- |
| GAPDH | TTATGACCACTGTCCACGCC | TCAGATCCACAACGGACACG | Ovine |
| IFNα | AAGACTTCGCATTCCCCCAG | GCTTGTCCAGGAGGATCTCG |  |
| IFNβ | TTCCAACAAGGACGGAGCCTT | GAACTGCTGTGCTTGCTTCAT |  |
| IFNγ | GGCCGGAGAGCATCAATGTA | GCCAAGTTGGACCCTGAGAT |  |
| ISG15 | ACATCCTGGTGAGGAACGAC | ATCCATGGGCTTCCCTTCAA |  |
| ISG20 | TCCGTGCTGTACGACAAGTT | TGTCGTAGATGGCGTAGCTG |  |
| IFITM1 | ACTCCTGGGTAAAGGAGGGTC | GGGCCCCGGTGAATAAGAG |  |
| OAS2 | CAAAGGCGGCTCCTATGGC | CACCTAAGCCCAGAGGGTTG |  |
| MX1 | GAAGAGCCTGCTGTGGACAT | GGTCCTGGCAGTAGACCAAC |  |
| IL1β | GAAGCTGAGGAGCCGTGCCTACGAACA | CCAGCACCAGGGATTTTTGCTCTCTGTCC |  |
| IL2 | AAACCTGAACACCAGAGAGAT | GCCTTTACTGTCGCATCA |  |
| IL4 | CCAAAGAACGCAACTGAGAA | GCTGCTGAGATTCCTGTCAA |  |
| IL5 | CATCTGCGTTTGACCTTGG | AGTTCCCATCACCTATCAGCA |  |
| IL6 | CGAGTTTGAGGGAAATCAGG | GTCAGTGTGTGTGGCTGGAG |  |
| IL8 | ATTCCACACCTTTCCACCCC | TGGGGTCTAAGCACACCTCT |  |
| IL10 | TCGTATGCCAATGCCCTCA | GATGAGGTAAAGCCCGTCAGT |  |
| CCL2 | CGCTCAGCCAGATGCAATTA | GTCCTGGACCCATTTCAGGT |  |
| CCL3 | CCTGCTGCTTCTCCTATGC | TGGAAGATGACACCAGGCTT |  |
| CCL4 | TCCTCGCAGCTTTGTGATTG | TCAGTTCGAGGTCATCCATGT |  |
| CCL5 | CCATGGCAGCAGTTGTCTTT | CACCCACTTCTTCTCTGGGT |  |
| CCL20 | CTCCTGGCTGCTTTGATGTC | ATGTCACAGGCTTCATTGGC |  |
| CXCL10 | ATACACGCTGTACCTGCATC | TGTGGCAATAATCTCGACACG |  |
| TNFα | TCGTATGCCAATGCCCTCA | GATGAGGTAAAGCCCGTCAGT |  |
| GAPDH | TCATCATCTCTGCCCCTTCT | GTCATGAGTCCCTCCACGAT | Procine |
| ISG15 | AGCATGGTCCTGTTGATGGTG | CAGAAATGGTCAGCTTGCACG |  |
| gB | GTCCGTGAAGCGGTTCGTGAT | ACAAGTTCAAGGCCCACATCTAC | PRV |

Table S2 siRNA sequences used for RNA interference

| **SiRNA** | **Sense (5’-3’)** | **Antisense (5’-3’)** | **Species** |
| --- | --- | --- | --- |
| ISG15 | GACCAUUUCUGGCUGACUUTT | AAGUCAGCCAGAAAUGGUCTT | Procine |
| scrambled control | UUCUCCGAACGUGUCACGUTT | ACGUGACACGUUCGGAGAATT |  |

**Supplemental Figures**


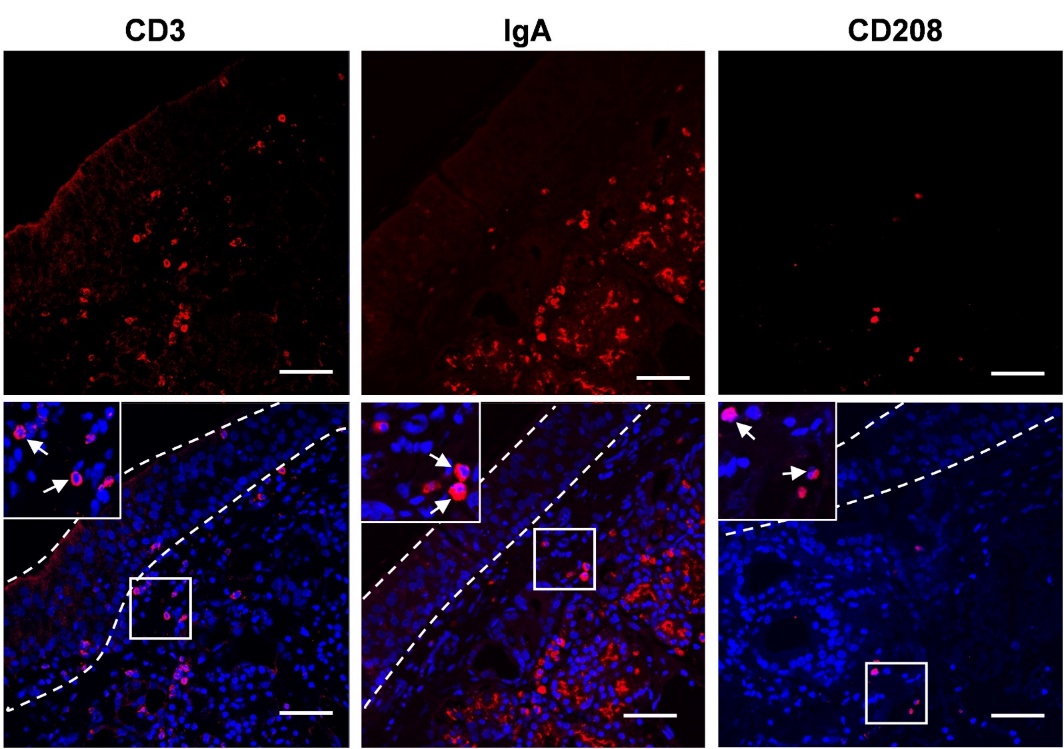


**Figure. S1 Detection of immunocyte of sheep nasal mucosa explants**

Immunofluorescence staining was used to detect the CD3, IgA and CD208 positive cells of sheep nasal mucosa explants. White arrows represent positive cells. The nasal mucosa explants were obtained from three mucosal tissues of three sheep. Scar bar = 50 μm.


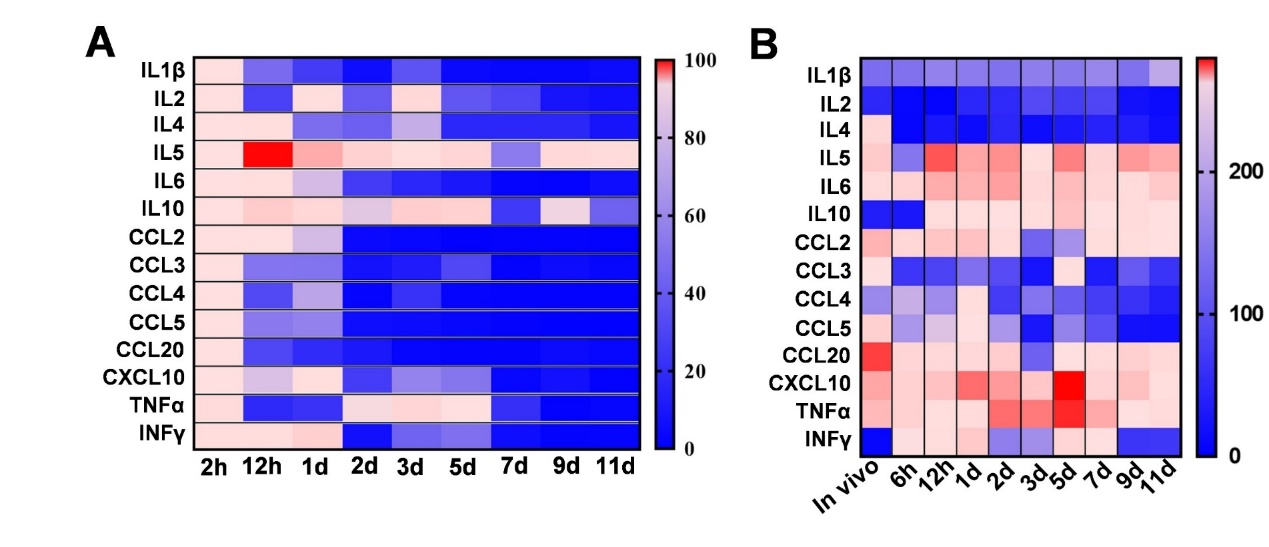


**Figure. S2 Characteristics of expression levels of innate immune factors in sheep**

**nasal mucosa explants**

The heatmap showed the expression characteristics of inflammatory factors in sheep nasal mucosa explants at different culture times, including horizontal and vertical comparison. The nasal mucosa explants of sheep at different times were obtained from three mucosal tissues of three sheep.


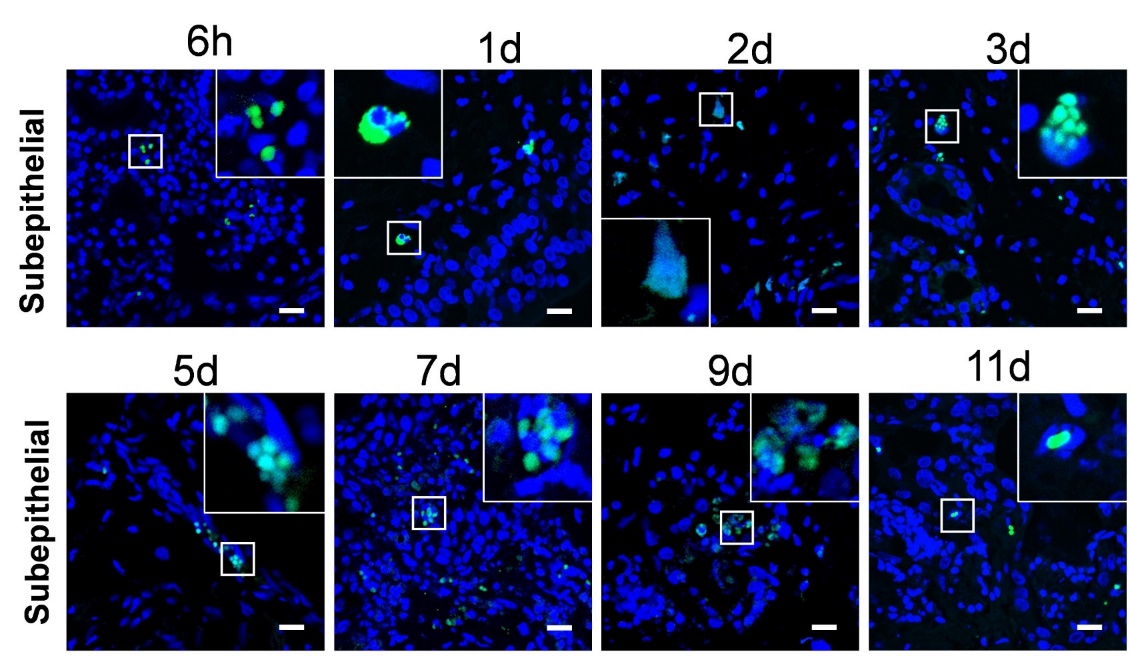


**Figure. S3 Detection of apoptosis of sheep nasal mucosa explants**

Tunnel kit was used to detect the apoptosis of sheep nasal mucosa explants in the subepithelial at different time points (6 h, 1 d, 2 d, 3 d, 5 d, 7 d, 9 d and 11 d). The nasal mucosa explants of sheep at different times were obtained from three mucosal tissues of three sheep. Scar bar = 20 μm.


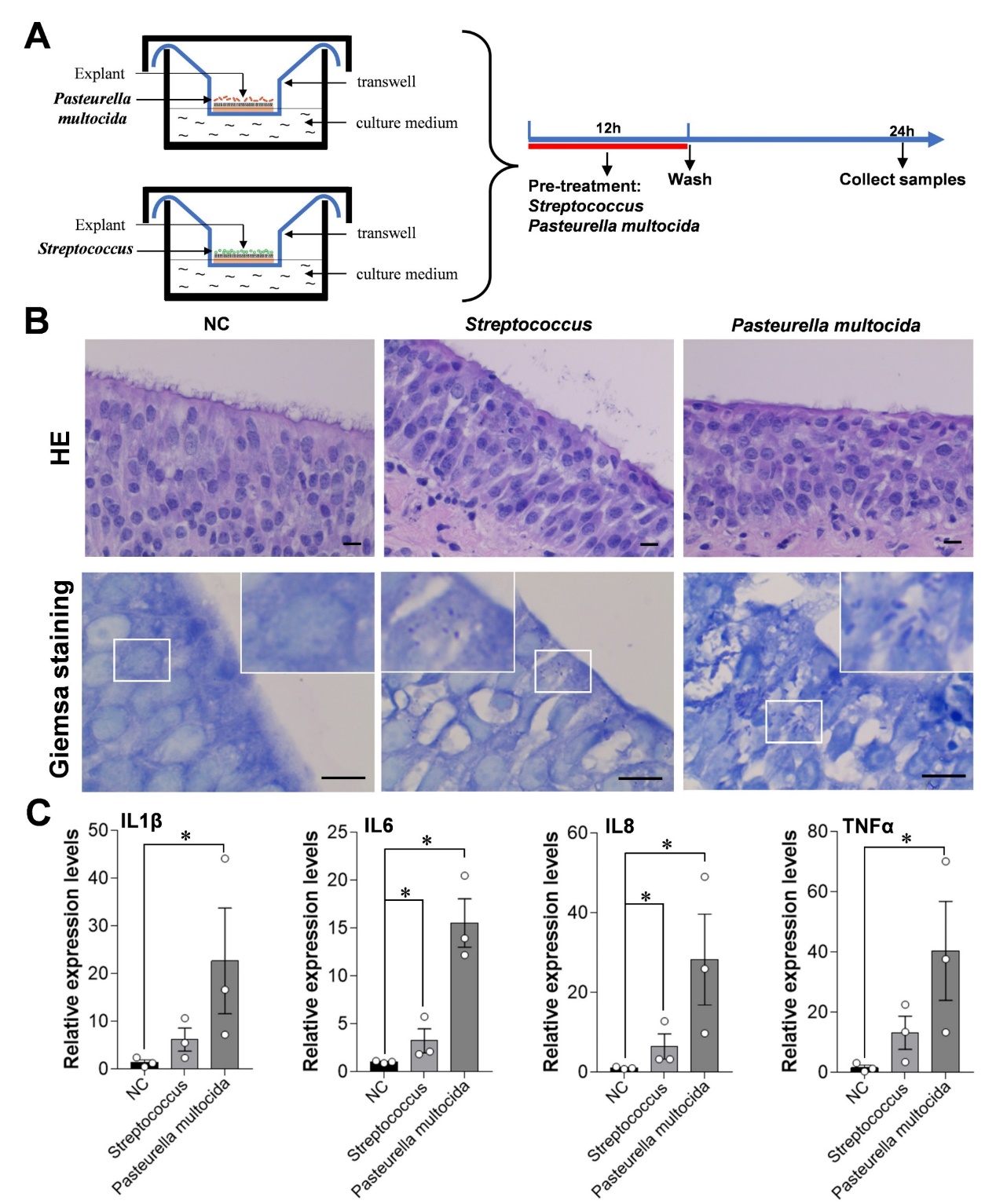


**Figure. S4 Characteristics of sheep nasal mucosa explants infected by *Streptococcus* and *Pasteurella multocida***

(A) The nasal mucosa of sheep was pretreated with *Streptococcus* and *Pasteurella multocida* for 12 h, and samples were collected at 24 h. (B) HE and Giemsa were used evaluate of the structure characteristics. (C) Detection of the changes of inflammatory factors after *Streptococcus* and *Pasteurella multocida* treatment by qRT-PCR. Sheep nasal mucosa explants from three replicates of the same sheep. Scar bar = 10 μm.


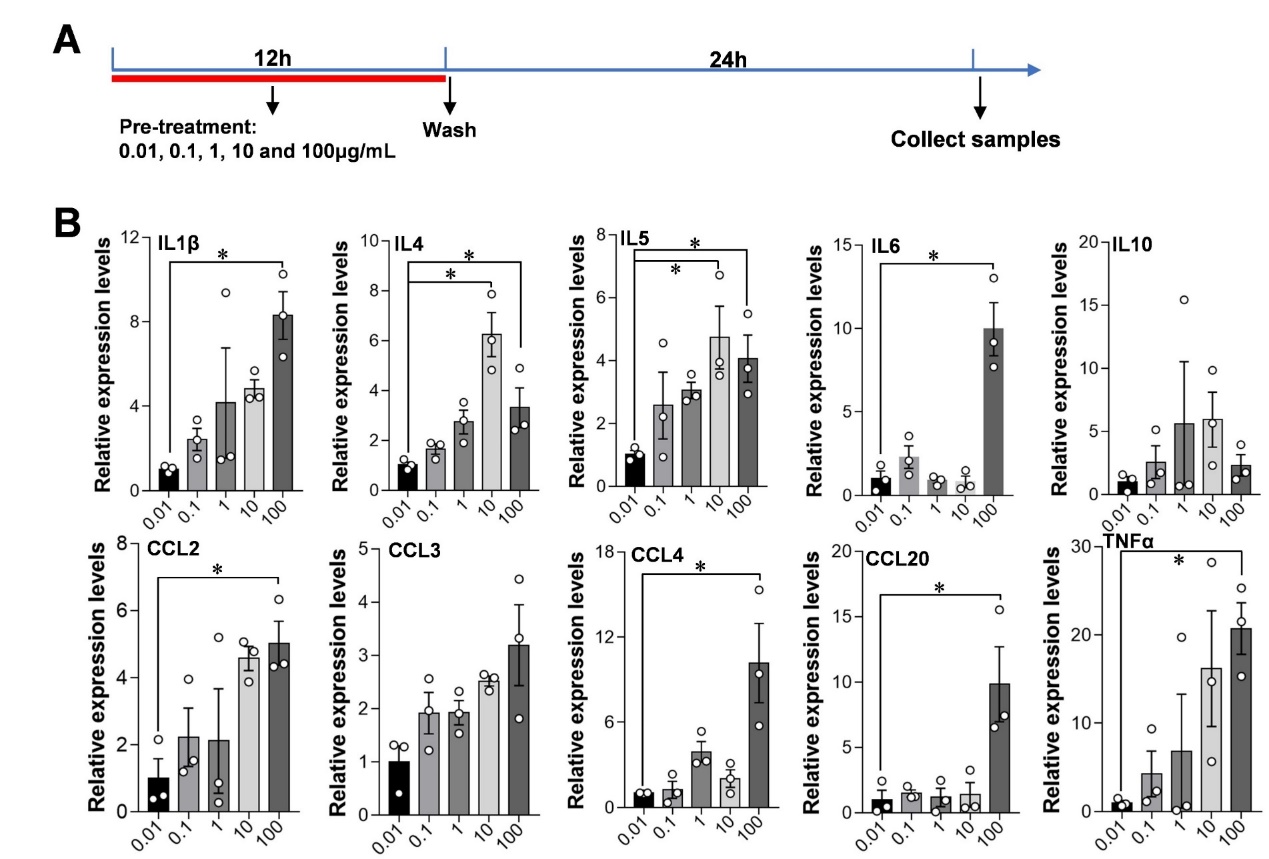


**Figure. S5 Characteristics of LPS induced sheep nasal mucosal explant inflammation**

(A) The nasal mucosa of sheep was pretreated with different concentrations of LPS (0.01, 0.1, 1, 10 and 100 μg/mL) for 12 h, and samples were collected at 24 h. (B) Detection of the changes of inflammatory factors after LPS treatment with different concentrations by qRT-PCR. Sheep nasal mucosa explants from three replicates of the same sheep.


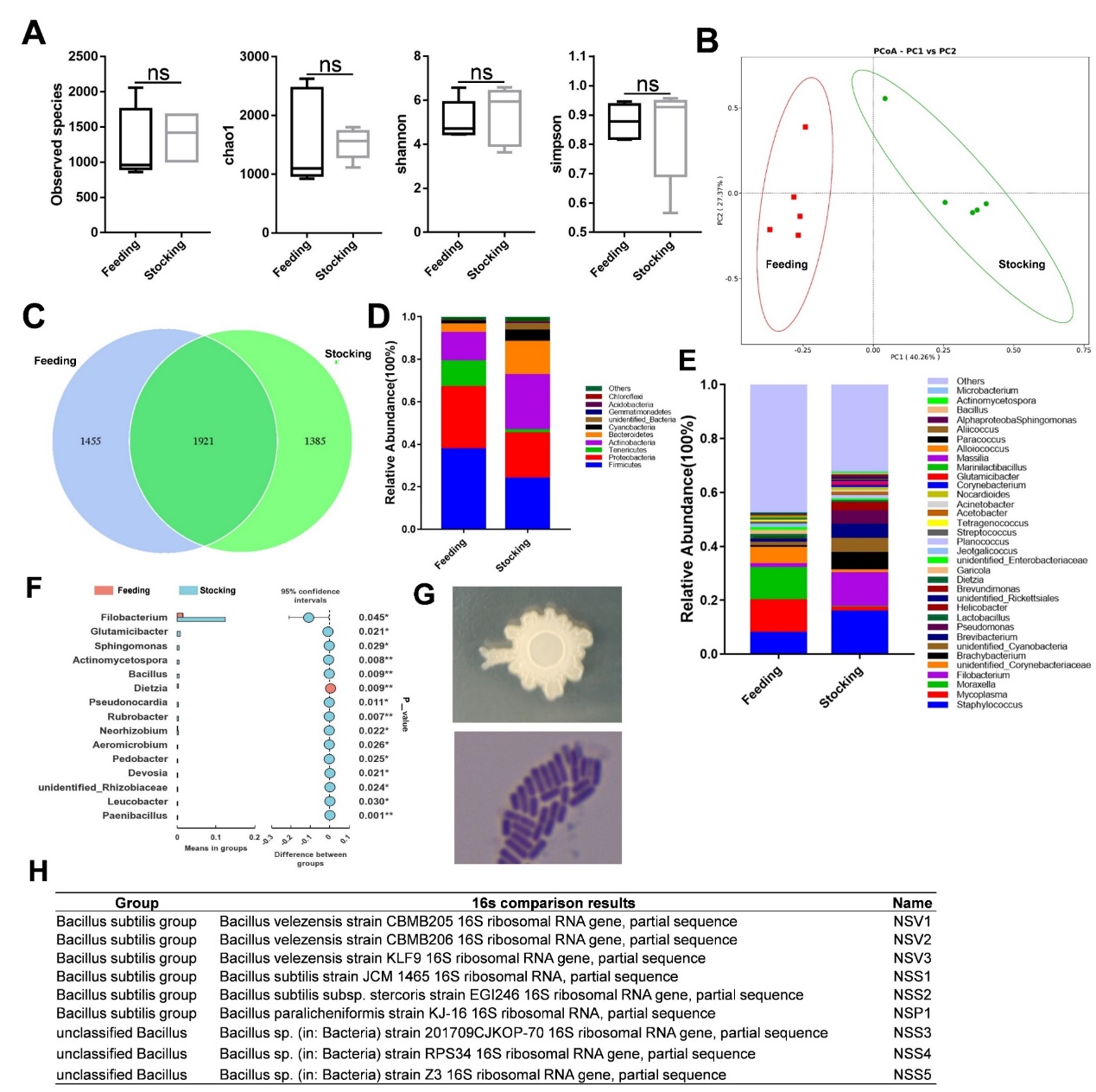


**Figure S6 Isolation and identification of *Bacillus subtilis* from sheep nasal cavity**

(A) Comparison of alpha diversity (Observed species, Chao1, Shannon index, and Simpson’s index) between the nasal microbiome of the Socking and Feeding nasal swab samples. (B) PCoA shows the bacterial composition clustering of the Socking and Feeding group based on Bray–Curtis distances, with each point corresponding to a sample and colored according to the sample type. Based on the rank of Bray Curtis distance value, the significance test of the difference between groups was carried out. If R-value is greater than 0, it indicates that the difference between groups is significant, and *p* < 0.05 indicates statistical significance. (C) Venn diagram showed the shared and unique OTUs in nasal swab samples collected from Socking and Feeding groups. (D and E) The relative abundance of bacteria in the nasal middle gate (top10) and the genus level (top35) of the stocking and feeding. (F) The comparison of the genus level flora with significant difference in nasal cavity between stocking and feeding sheep. The p-value was calculated by Welch's t-test. (G) *Bacillus subtilis* colony isolated and was observed by Gram staining. (H) Statistics of isolated *Bacillus subtilis* group strains.

**Supplemental video**
